# Editorial: Renal physiology: epithelial cell mechanics

**DOI:** 10.3389/fphys.2024.1428778

**Published:** 2024-06-04

**Authors:** Francesca Di Sole, Kamel Laghmani, Victor Babich

**Affiliations:** ^1^ Physiology and Pharmacology Department, Des Moines University, Des Moines, IA, United States; ^2^ INSERM U1138, Centre National de la Recherche Scientifique, ERL8228, Paris, France; ^3^ Department of Liberal Arts and Sciences, Mercy College of Health Sciences, Des Moines, IA, United States

**Keywords:** energy balance, lipid accumulation, acid-base balance, polycystic kidney disease, primary cilium, acute kidney injury, diabetic kidney disease

Epithelial cells form the outer layer of the renal tubules within the nephron, possessing mechanical properties that are essential for maintaining normal kidney function. Disruption of epithelial cell mechanics can contribute to the pathogenesis of kidney disorders. Hence, understanding the mechanics of renal epithelial cells is essential for unraveling the pathophysiology of kidney diseases and developing new therapeutic strategies. This highlights the importance of studying the mechanical properties of renal epithelial cells in both health and disease.

This Research Topic provided some highlights on key themes in epithelial cell mechanics with seven articles selected for publication from a total of 12 submitted manuscripts. All selected articles are authored by expert contributors who have given a prospective on how recent data have enriched the understanding of the mechanical properties of renal epithelial cells in the context of energy balance, fluid and electrolyte transport and primary cilium function in health and disease.

Dysregulation of renal epithelial cell mechanics in the context of energy balance and lipid accumulation contributes to the pathogenesis of obesity-related kidney diseases, such as diabetic nephropathy, obesity-related glomerulopathy, and non-alcoholic fatty liver disease (NAFLD)-associated nephropathy. For example, mitochondrial-cytoskeletal interactions have a well-established role in mitochondrial motility. These interactions also regulate the balance of mitochondrial fission/fusion, as well as mitochondria turnover (Moore and Holzbaur, 2018). In acute kidney injury (AKI), surviving cells change by dedifferentiating, migrating, and proliferating. However, tubule cells often fail to properly re-differentiate after AKI, leading to atrophy. This is characterized by reduced and smaller mitochondria (Chen et al., 2024). Understanding how energy metabolism and cell mechanics interact is crucial for developing new treatments for kidney diseases. Wenbin Tang and Qingqing Wei reviewed in their manuscript metabolic impairment induced by renal injury and reprogramming in renal repair. The authors specifically discussed how kidney repair after AKI is featured by metabolic reprogramming, which regulates tubular degeneration, proliferation, and differentiation. This reprogramming includes the suppression of fatty acid β-oxidation, citric acid cycle, and oxidative phosphorylation, along with the induction of glycolysis. The dysregulation of pentose phosphate pathway in AKI was also discussed in this review article in conjunction with the therapeutic potential of targeting these metabolic pathways as new avenues for intervention.

Related to this research topic, Rong et al. investigated the effect of berberine on abnormal renal lipid accumulation in renal tubular epithelial cells, which contributes to cellular lipotoxicity and disturbed mitochondrial bioenergetics in diabetic kidney disease. Berberine, an isoquinoline alkaloid and a major active constituent of *Rhizoma coptidis* (Huanglian in traditional Chinese Medicine) and *Cortex phellodendri* (Huangbai in traditional Chinese Medicine)*,* was studied to assess its impact on the development of lipotoxicity and tubulointerstitial fibrosis contributing to the progression of diabetic kidney disease. The study demonstrates that berberine treatment alleviates glomerular sclerosis and attenuates tubulointerstitial injury in type 2 diabetic mice. Additionally, it was found to suppress renal tubular epithelial cell injury by reducing lipid deposition and preserving mitochondrial function.

Epithelial cell mechanics are also essential for fluid transport in the nephron and for detecting mechanical signals like fluid flow and pressure changes. Renal epithelial cells, with structures like primary cilia, act as mechano-sensors to sense and transmit signals regulating renal functions such as the maintenance of salt and acid-base homeostasis.

Preserving acid-base balance is vital for normal body function, with the kidneys playing a fundamental role. In the study by Milano et al., β3 adrenoceptors (β3-AR), localized in collecting duct intercalated cells, were found to impact acid-base regulation. The authors demonstrate that activation of β3-AR enhances H^+^-ATPase expression and activity, crucial for renal acid-base control. Sympathetic nerve stimulation affects renal function via tubular epithelial cell receptors such as adrenoreceptors and this study highlights β3-AR’s role in sympathetic regulation of renal acid-base balance.

Another essential component of pH regulation is the bicarbonate transport which maintains pH balance by eliminating excess acids or bases through urine or blood buffering systems. Bicarbonate transporters of the SLC4 family in the medullary thick ascending limb of the loop of Henle are important key players not only in renal acid-base homeostasis but also in sodium reabsorption regulation. Research by Cai et al. delves into how salt reabsorption is regulated in the medullary thick ascending limb via the SLC4 family of bicarbonate transporters. The data of the study strongly suggest that bicarbonate transporters NBCn1, NBCn2, and AE2 are upregulated during high salt intake via protein-protein interactions with the inositol 1,4,5-trisphosphate, providing, therefore, new insights into molecular mechanisms underlying sodium balance.

Furthermore, Liu et al. studied how potassium (K^+^) channel activity affects membrane potential in renal proximal tubule cells and its impact on salt and bicarbonate reabsorption. Adenosine receptor activation is known to influence renal tubule cell processes (Di Sole, 2008; Pak et al., 2022). This study specifically delves into adenosine regulation of basolateral K^+^ channels in the proximal tubule, along with the signaling pathway responsible for mediating adenosine’s effect on these channels.

Mutations in genes affecting cell-cell junctions or cytoskeletal components can impair tubular function, leading to renal disorders like polycystic kidney disease (PKD). Polycystin-2 (PC2), a calcium-permeable and nonselective cation channel, is involved in autosomal dominant polycystic kidney disease (ADPKD) and is found in renal epithelial cell cilia. The findings presented by Scarinci et al. suggest that factors influencing PC2 function or gene expression can alter the length of primary cilia in renal epithelial cells. The authors propose that regulating PC2 activity within the primary cilium may be crucial in initiating mechanisms that lead to cyst formation in ADPKD. In relation to the function of the primary cilium in renal epithelial cells, Scarinci et al. also propose that microtubules in the primary cilium generate electrical oscillations that, when synchronized and propagated, serve as a signaling mechanism. Additionally, the authors found that the deletion of PC2 alters the electrical behavior of microtubules, establishing a link between ciliary channels and the electrical dynamics of the cilium.

Overall, renal physiology epithelial cell mechanics play a critical role in maintaining normal kidney function and are implicated in various renal disorders, highlighting the importance of studying the mechanical properties of renal epithelial cells. Therefore, we believe that this Research Topic of articles will support future progress in the field, and we are grateful to the contributors and reviewers for their efforts on this Research Topic.
